# The functional role of lncRNAs as ceRNAs in both ovarian processes and associated diseases

**DOI:** 10.1016/j.ncrna.2023.11.008

**Published:** 2023-11-18

**Authors:** Muhammad Usman, Ai Li, Dan Wu, Yang Qinyan, Lin Xiao Yi, Guiqiong He, Hong Lu

**Affiliations:** a Department of Plastic and Reconstructive Surgery, Central Hospital Affiliated to Chongqing University of Technology, Gonglian yicun No.1 street lijiatuo, Banan district, Chongqing, 400054, PR China; b Department of Postdoctoral Research Workstation, The Seventh People's Hospital of Chongqing, Chongqing, PR China; c Clinical Medical Research Center, Southwest Hospital, Third Military Medical University (Army Medical University), Chongqing, PR China; d Department of Anesthesia, Central Hospital Affiliated to Chongqing University of Technology, Gonglian yicun No.1 street lijiatuo, Banan district, Chongqing, 400054, PR China; e Department of Radiology, The Chenjiaqiao Hospital of Shapingba District of Chongqing, PR China; f Institute of Neuroscience, Basic Medical College, Chongqing Medical University, Chongqing, 400016, PR China; g Department of Medical Imaging, Central Hospital Affiliated to Chongqing University of Technology, Gonglian yicun No.1 street lijiatuo, Banan district, Chongqing, 400054, PR China

**Keywords:** Long non-coding RNAs (lncRNAs), Gene expression, Ovarian function, Ovarian biology, Targeted therapies, Reproductive health

## Abstract

Long non-coding RNAs (lncRNAs) have attracted significant scientific attention due to their central role in regulating gene expression and their profound impact on the intricate mechanisms of ovarian function. These versatile molecules exert their influence through various mechanisms, including the coordination of transcription processes, modulation of post-transcriptional events, and the shaping of epigenetic landscapes. Furthermore, lncRNAs function as competitive endogenous RNAs (ceRNAs), engaging in intricate interactions with microRNAs (miRNAs) to finely adjust the expression of target genes. The intricate lncRNA-miRNA-mRNA network serves as a crucial determinant in governing the multifaceted physiological functions of the ovaries. It holds substantial potential in unraveling the causes and progression of reproductive disorders and, importantly, in identifying new therapeutic targets and diagnostic markers for these conditions. A comprehensive comprehension of lncRNAs and their ceRNA activities within the domain of ovarian biology could potentially lead to groundbreaking advancements in clinical interventions and management strategies. This exploration of lncRNAs and their intricate involvement in the regulatory framework provides an extensive platform for deciphering the complex nature of ovarian physiology and pathology. The ongoing progress in this field, which encompasses in-depth investigations into the functional roles of specific lncRNAs, the elucidation of their complex interactions with miRNAs, and the comprehensive profiling of their expression patterns, holds the promise of making significant contributions to our understanding of ovarian biology and reproductive disorders. Ultimately, these breakthroughs will have wide-ranging translational implications, paving the way for the development of precision therapies and personalized medicine strategies to address the myriad challenges in the realm of reproductive health.

## 1 Introduction

The ovaries, indispensable components of the female reproductive system, fulfill a dual role that encompasses both reproductive and endocrine functions. They serve as the primary source of egg production, a pivotal aspect of reproduction, and concurrently, they play a central role in the secretion of essential female hormones that govern various physiological processes. The successful execution of this dual role demands a highly intricate and finely tuned physiological process, where precise regulation of numerous cellular factors and the associated genes is imperative. In recent years, there has been a transformative shift in our comprehension of long non-coding RNAs (lncRNAs). These molecules were previously relegated to the status of mere “noise” within the vast transcriptome. However, thanks to the advent of cutting-edge sequencing technologies, we have come to recognize their profound significance. LncRNAs have emerged as pivotal players in the intricate orchestra of gene expression regulation, showcasing their capacity to influence protein function at multiple levels, ranging from transcription to post-translational modification [[1], [2], [3], [4]]. The world of lncRNAs is an intricate one, characterized by a diverse array of mechanisms through which they exert their regulatory influence. Their role as competing endogenous RNAs (ceRNAs) has garnered substantial attention in the intricate web of regulatory interactions that govern ovarian function and the diseases associated with it [5]. Despite the burgeoning interest and growing recognition of the pivotal role that lncRNAs play in these contexts, many facets of their specific functions within the ovaries and the intricate details of their regulatory roles as ceRNAs in ovarian function remain enigmatic. Thus, there is a pressing need for a more profound and comprehensive exploration of this captivating realm of scientific inquiry.

## 2 CERNA network

CeRNAs form a unique category of RNA molecules that share common microRNA (miRNA) binding sites known as miRNA response elements (MREs). These ceRNAs engage in competitive interactions, effectively vying for the opportunity to bind to MREs. This competitive dynamic leads to the modulation of miRNA levels, thereby exerting a significant influence on the miRNA-mediated control of target genes. In essence, they act as mediators of communication and play a crucial role in post-transcriptional regulation, facilitating the exchange of information among a diverse array of RNA species. The concept of the ceRNA regulatory network, initially proposed by Salmena et al., in 2011, has been heralded as a groundbreaking “new language” for understanding the intricate web of RNA interactions within the realm of transcriptomics. This concept unveils a complex network of ceRNAs, encompassing both coding and non-coding RNAs, all actively participating in competitive interactions with miRNAs. The result is the formation of an expansive and interconnected network that maintains a dynamic equilibrium. When specific RNA molecules within this network experience dysregulation, it can disrupt the delicate balance and give rise to corresponding aberrations in biological organisms. At the core of the ceRNA regulatory network lie miRNAs, which are endogenous, non-coding small RNAs typically composed of approximately 20–24 nucleotides. MiRNAs primarily engage with target gene molecules through sequence-specific interactions, leading to the inhibition of translation or the degradation of these target genes [6]. This regulatory process exerts a negative influence on the expression of target genes at the protein level. RNA molecules that actively compete for miRNA binding, thereby influencing the expression levels of target genes, are termed ceRNAs. This category encompasses a diverse range of RNA species, including long non-coding RNAs (lncRNAs), circular RNAs (circRNAs), mRNAs, and others. In the context of the ceRNA regulatory network, lncRNAs have garnered significant attention in recent years due to their critical role in maintaining normal ovarian function and influencing reproductive endocrine health [7,8]. They represent a vital component of the intricate puzzle of ceRNA interactions, greatly enhancing our understanding of gene regulation within the field of female reproductive biology.

## 3 LNCRNA

**Fig. 1:**
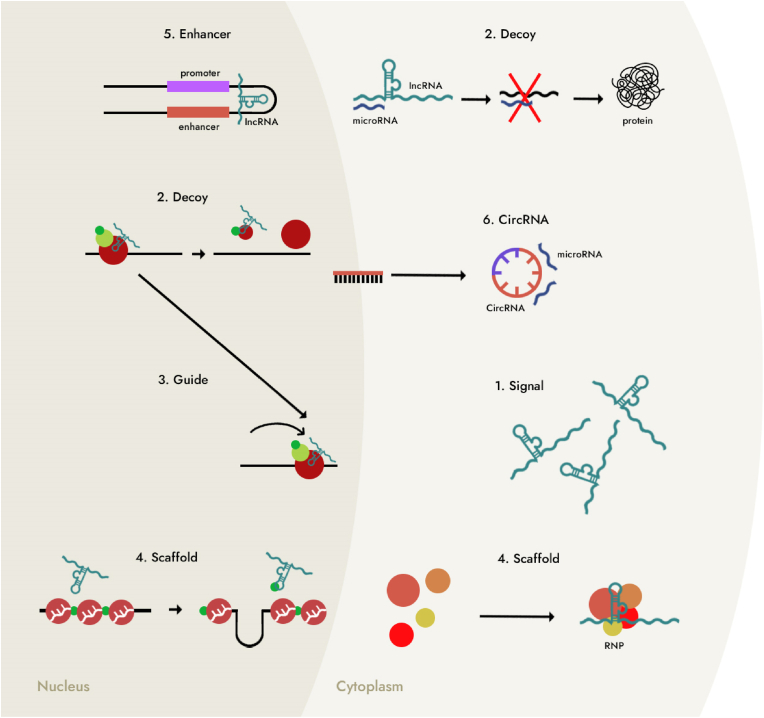
Presents the various functions attributed to long noncoding RNAs (lncRNAs). These functions can be categorized as follows: Signal lncRNAs: These lncRNAs are expressed at specific time points and in specific cellular locations, indicating their involvement in temporal and spatial signaling events. Decoy lncRNAs: These lncRNAs could bind to regulatory factors and microRNAs, altering their normal function and potentially modulating gene expression. Guide lncRNAs: These lncRNAs play a role in regulating gene activation or repression by facilitating the relocation of ribonucleoprotein complexes within the cell. Scaffold lncRNAs: Like guide lncRNAs, scaffold lncRNAs also facilitate the formation of ribonucleoprotein complexes; however, they primarily affect the molecular components of the complex itself. Enhancer lncRNAs: Produced from enhancer elements, these lncRNAs influence the activation of target genes by modulating enhancer-promoter interactions. Circular lncRNAs: Circular lncRNAs can interfere with various RNA processing and splicing events, and they can also act as sponges for microRNAs, sequestering them and preventing their regulatory functions.

LncRNAs, which stand for long non-coding RNAs, represent a significant and diverse category within the realm of non-coding RNAs (ncRNAs). What distinguishes them is their substantial length, often exceeding 200 nucleotides. Remarkably, lncRNAs constitute a major portion of the ncRNA landscape, accounting for a substantial percentage, typically ranging from 80 % to 90 % of all ncRNAs [9]. Their abundance underscores their importance in cellular processes. LncRNAs exhibit notable diversity, primarily based on their genomic origins and locations. They can be classified into five principal categories: sense, antisense, intergenic, intronic, and bidirectional lncRNAs [10]. These categories reflect the various genomic contexts from which lncRNAs originate, further highlighting their multifaceted nature. Research has illuminated the involvement of lncRNAs in a wide array of epigenetic processes. These include histone and chromatin modifications, which play critical roles in shaping the structure of DNA and gene expression. LncRNAs also contribute to genomic imprinting, a process essential for regulating gene expression from parental alleles [11,12]. Their influence extends to the transcriptional level, where lncRNAs impact mRNA production. They achieve this by modulating the activity of specific transcription factor complexes, essentially shaping the transcriptional landscape of the cell [13]. Beyond transcriptional control, lncRNAs have a significant role in post-transcriptional gene regulation. They participate in processes such as splicing, processing, transport, translation, and degradation of RNAs, thereby fine-tuning gene expression post-transcriptionally [14]. The multifaceted involvement of lncRNAs across both transcriptional and post-transcriptional domains underscores their pivotal role in orchestrating gene expression at various levels. LncRNAs can be broadly categorized into three groups based on their mechanisms of action. Firstly, they operate at the chromatin level, where they exert their influence by inducing epigenetic changes to the chromatin structure, ultimately impacting gene expression. Secondly, at the transcriptional level, lncRNAs regulate gene expression by modifying the organization of transcriptional machinery and influencing the activity of associated factors and enzymes. This level of control significantly shapes the transcriptional output of the cell. Thirdly, at the post-transcriptional level, lncRNAs engage in intricate interactions with mRNAs and miRNAs. This interplay allows them to modulate the expression levels of these crucial RNA molecules. These three broad categories of action emphasize the versatile and multifaceted roles that lncRNAs play in the complex landscape of gene regulation (see Fig. 1) [15]. LncRNAs, therefore, emerge as key players in the intricate symphony of genetic control within the cell, influencing processes critical for cellular function and development.

Thousands of lncRNAs have been identified in mammals and other vertebrates, with some extensively studied [15].

## 4 LNCRNA regulates ovarian physiological function

Certainly, let's delve even deeper into the pivotal role of long non-coding RNAs (lncRNAs) in the regulation of ovarian physiological functions, emphasizing their intricate involvement in both reproductive and endocrine aspects [16]:

### 4.1 Orchestrating ovarian reproductive function

LncRNAs emerge as central conductors in the complex symphony of processes within the ovaries that directly impact reproductive function. Their influence spans a spectrum of critical roles, from steering the development of granulosa cells to shaping the course of oocyte maturation. They also play a significant part in the regulation of ovulation and are instrumental in guiding the formation of the corpus luteum. At the core of ovarian function lies the ovarian follicle, where a central oocyte is enveloped by a cohort of granulosa cells. The meticulous development and maturation of these follicles are choreographed dances, necessitating precise interactions between the oocyte, granulosa cells, and theca cells. Any discord in these intricately orchestrated events can give rise to abnormal follicle development, contributing to a myriad of reproductive challenges. The corpus luteum, formed through the transformation of luteinized granulosa and theca cells after ovulation, stands as a linchpin for successful embryo implantation. Adequate luteal function is directly intertwined with fertility, and disruptions can lead to recurrent miscarriages. LncRNAs, through their elaborate and multifaceted regulatory mechanisms, assume a pivotal role in maintaining the delicate equilibrium of these essential reproductive processes, influencing the ability to conceive and sustain a pregnancy to term.

### 4.2 Guardians of ovarian endocrine function

Going beyond their roles in reproductive functions, the ovaries serve as essential endocrine hubs responsible for the synthesis and secretion of pivotal female hormones. These hormones encompass estrogen, progesterone, and androgens, as well as anti-Müllerian hormone (AMH). The regulation of these hormones is paramount for a multitude of endocrine functions within the female body, extending their influence over the menstrual cycle, pregnancy, and overall endocrine well-being. LncRNAs actively participate in the intricate choreography of hormone synthesis and secretion, wielding their influence as master regulators of the endocrine balance. Their impact ripples through the complex hormonal interplay that governs the entirety of female physiology, from the onset of puberty to the transition into menopause.

In essence, lncRNAs emerge as pivotal architects in the intricate tapestry of ovarian physiology. Their multifaceted involvement in both reproductive and endocrine dimensions underscores their paramount significance in ensuring the proper functioning of the ovaries. A comprehensive grasp of the myriad roles lncRNAs play in ovarian regulation is indispensable for advancing our understanding of female reproductive biology. It also lays the foundation for potential therapeutic interventions aimed at addressing reproductive disorders and enhancing women's health.

### 4.3 Involved in the development of granulosa cells

The proliferation and maturation of granulosa cells are pivotal for the proper development of ovarian follicles, and disruptions in these processes can have significant repercussions for reproductive function.

**Fig. 2:**
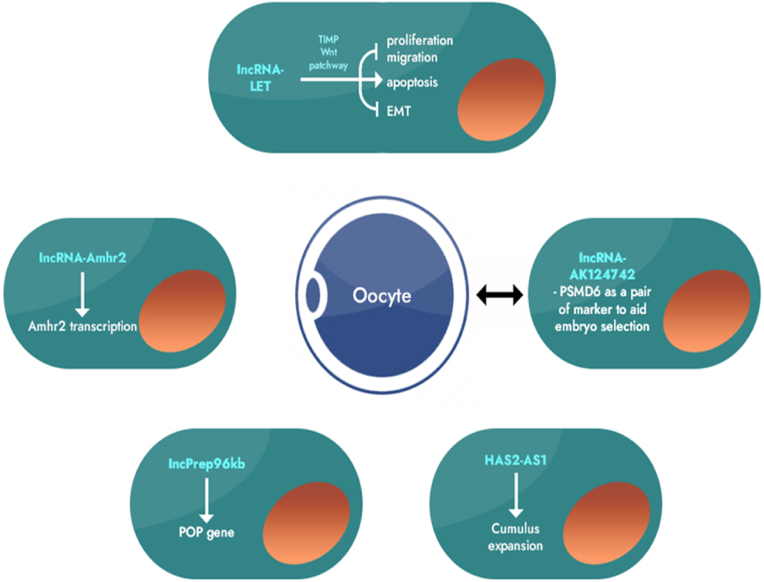
Summarizes the roles of specific lncRNAs in normal granulosa cells (GCs). lncRNA-LET inhibits proliferation, migration, and EMT while promoting apoptosis in KGN GCs. lncRNA-Amhr2 activates Amhr2 in primary mural and cumulus GCs and OV3121 cells. lncRNA-lncPrepþ96kb induces POP in ovarian GCs. AK124742-PSMD6 is a potential lncRNA-mRNA biomarker in cumulus GCs for embryo selection. HAS2-AS1 activates HAS2 and induces cumulus GCs migration.

A study conducted by York and colleagues, employing genetically engineered mouse models, unveiled the critical role of the long non-coding RNA Growth Arrest Special-Transcript 2 (GAS2) in the ovaries of mice [17]. Their findings illuminated the significance of GAS2 in regulating the Notch signaling pathway, a vital pathway governed by Notch genes. The ramifications of this disruption are profound, encompassing a reduction in reproductive capacity in mice, cystic rupture of oocytes, a decrease in the numbers of antral follicles and corpora lutea, and severe damage to the basal membrane tissue surrounding growing follicles. This intricate disruption ultimately hampers the development of follicles (Fig. 2).

Another study delves into the intricate molecular mechanisms underlying bovine follicular cysts, a significant challenge in the bovine breeding industry [18]. Bovine ovarian cysts have not only been a persistent scientific puzzle but also a source of substantial economic losses. By exploring the interplay of non-coding RNAs (ncRNAs) and competitive endogenous RNA (ceRNA) networks, this research strives to unravel the pathogenesis of bovine follicular cysts. The study aptly highlights the paramount importance of understanding and addressing bovine follicular cysts in the context of the bovine breeding industry. These cysts have been elusive challenges with far-reaching economic consequences. The study adopts a comprehensive approach by conducting whole transcriptome sequencing of bovine follicular granulosa cells (GCs). This approach provides valuable insights into the expression profiles of mRNAs, long non-coding RNAs (lncRNAs), and microRNAs (miRNAs) in cystic and normal follicular GCs. The research uncovers significant alterations in the expression patterns of 8,003 mRNAs, 579 lncRNAs, and 205 miRNAs between cystic and normal follicular GCs. This indicates a robust difference in the genetic and regulatory landscapes of these cells. To gain a deeper understanding of the implications of these differential expressions, the study conducts Gene Ontology and Kyoto Encyclopedia of Genes and Genomes pathway analyses. This provides insights into the biological processes and pathways affected by these expression changes. The research extends its investigation to the realm of ceRNA networks, connecting mRNAs, miRNAs, and lncRNAs. This approach employs co-expression analysis and bioinformatics methods, shedding light on the complex regulatory interactions between these RNA molecules. The study reveals a specific ceRNA pathway involving lncRNA NONBTAT027373.1, miR-664b, and HSD17B7. This pathway is validated through dual-luciferase reporting assays and RNA binding protein immunoprecipitation (RIP) assays. It underscores the role of lncRNAs in modulating miRNA activity and affecting gene expression. The findings emphasize the relevance of genes and lncRNAs associated with steroid hormone synthesis and energy metabolism in the formation of bovine cystic follicles. This insight carries significant implications for promoting healthy and efficient development in the bovine industry. This study offers valuable insights into the molecular underpinnings of bovine follicular cysts, a longstanding challenge in bovine breeding [18]. By uncovering the regulatory roles of non-coding RNAs and the intricate ceRNA networks, the research provides a foundation for further investigations and potential interventions in the bovine industry. The identification of candidate targets related to steroid hormone synthesis and energy metabolism marks a significant step toward addressing this complex issue.

Another study focuses on a crucial yet understudied aspect of long noncoding RNA (lncRNA) function in reproductive biology – its role in regulating hormone synthesis in ovarian follicular granulosa cells [19]. By narrowing down this area of research, the study fills an important gap in our understanding of reproductive mechanisms. The research builds upon the foundation of previous studies that demonstrated the role of lncRNA Gm2044 in promoting estradiol synthesis in follicular granulosa cells. This not only underscores the importance of replicating and extending previous findings but also hints at the potential clinical significance of this lncRNA. Using a sophisticated approach, the study identifies 21 binding proteins of lncRNA Gm2044 in ovarian follicles, further characterizing the interactions underlying its function. This mass spectrometry-based approach (ChIRP-MS) provides valuable insights into the molecular players involved. The research validates the interaction between lncRNA Gm2044 and eukaryotic translation elongation factor 2 (EEF2) protein through RNA immunoprecipitation (RNA IP) and reverse transcription PCR (RT-PCR). This confirmation strengthens the reliability of the findings. The study employs a lncRNA Gm2044 knockout mouse model created using the CRISPR/Cas9 method. This in vivo approach allows for the investigation of the lncRNA's impact on follicular development and fertility, as well as its influence on estradiol levels. The observation that estradiol concentration decreased in the knockout mice suggests a significant role for lncRNA Gm2044. The study goes further to decipher the molecular mechanisms by which lncRNA Gm2044 operates. It reveals that lncRNA Gm2044 promotes the binding of EEF2 to Nr5a1 mRNA, enhancing Nr5a1 mRNA translation. This, in turn, leads to increased estradiol synthesis, shedding light on a specific pathway of regulation. To explore the broader implications of lncRNA Gm2044, the research conducts transcriptome sequencing on ovaries of lncRNA Gm2044 knockout mice. This comprehensive analysis identifies significant gene expression changes and enrichments in Gene Ontology (GO) and Kyoto Encyclopedia of Genes and Genomes (KEGG) pathways, providing a more comprehensive view of its regulatory role. The study concludes by highlighting the importance of understanding how lncRNA Gm2044 and EEF2 protein influence estradiol synthesis. It suggests that this knowledge could be instrumental in the treatment of estrogen-related reproductive diseases, pointing to potential clinical applications. This research presents a significant contribution to the field of reproductive biology. By delving into the specific role of lncRNA Gm2044 in estradiol synthesis and unraveling the associated molecular mechanisms, it not only expands our understanding of reproductive processes but also hints at its potential clinical relevance.

Another key protagonist in ovarian function is the serine protease Prolyl Oligopeptidase (PREP), which finds high expression in granulosa cells. The knockout of the long non-coding RNA known as lncRNA-Prept+96 kb in female mice culminates in a diminished expression of prolyl oligopeptidase within granulosa cells, thereby resulting in impaired proliferation of these pivotal cells [20].

Furthermore, research has brought to light the role of the long non-coding RNA LINC-01572:28, which intricately interacts with the S-phase kinase-associated protein, consequently exerting inhibitory effects on the proliferation and cell cycle progression of ovarian granulosa cells [21].

While these discoveries underscore the substantial influence of long non-coding RNAs on the development of ovarian granulosa cells, the precise mechanisms through which they exert their effects remain a subject of ongoing research. To fully unlock the potential of these findings and harness their therapeutic implications for addressing reproductive disorders and improving female reproductive health, further clinical and experimental investigations are imperative. The intricate interplay between long non-coding RNAs and granulosa cells holds the promise of providing valuable insights into female reproductive biology, potentially leading to the development of targeted interventions. This burgeoning field carries the potential to significantly enhance our understanding of and ability to address reproductive challenges.

### 4.4 Involved in the maturation of oocytes

**Fig. 3:**
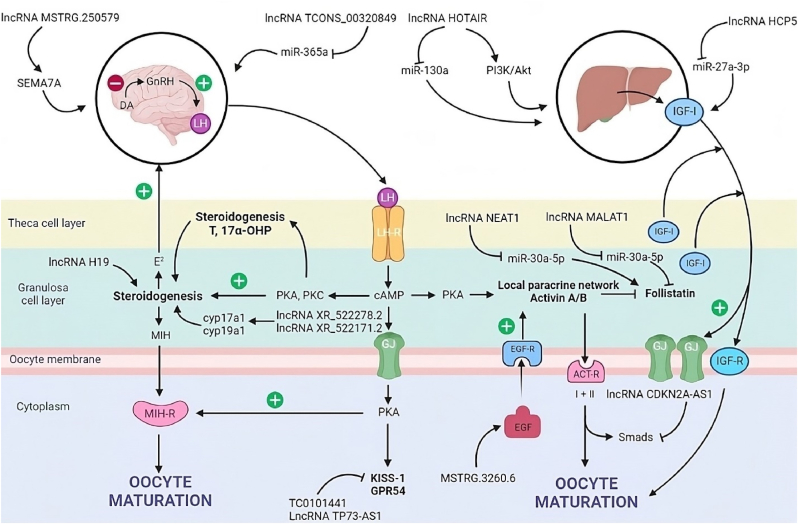
Schematic illustration of mechanisms of oocyte maturation and related long non-coding RNAs (lncRNAs) regulation. In humans and animals, many factors have been identified, including epigenetic molecules like lncRNAs and their various signaling pathways, that are critical for oocyte maturation. They not only regulate oocyte maturation, but also coordinate each other (balance) to ensure physiological conditions. For instance, gonadotropin hormone-releasing hormone (GnRh) and insulin-like growth factor (IGF) system influences important elements of reproductive system development, including oocyte maturation. IGF-1 is a polypeptide hormone produced mainly by the liver which stimulates GnRH release directly from neuroendocrine brain regions. IGF-1/GnRh system is under the control of the regulatory activities of lncRNAs and their downstream signaling pathways, providing a balance for normal oocyte development/maturation. Note: SEMA7A, Semaphorin 7A; PI3K, Phosphoinositide 3-kinases; Akt, serine/threonine kinase family; LH, Luteinizing hormone; 17α-OHP, 17α-Hydroxyprogesterone; MIH-R, Maturation-inducing hormone receptor; MIH, Maturation-inducing hormone; LH-R, Luteinizing hormone receptor; GJ, Gap junctions; IGF-R, Insulin-like growth factor type 1 receptor; PKA, Protein kinase A; PKC, Protein kinase C; cAMP, Human cathelicidin antimicrobial peptide; PKA, Protein kinase A; EGF-R, Epidermal growth factor receptor; EGF, Epidermal growth factor; DA, Dopamine.

The maturation of oocytes is a critical process in follicular development, closely tied to female reproductive capacity and the ability to undergo fertilization post-ovulation. Yerushalmi and colleagues made an intriguing discovery, noting that integrin alpha 6 antisense RNA (ITGA6-AS) experiences significant upregulation in mid-pachytene oocytes, while its host gene ITGA6 is downregulated [22]. Integrin α6β1 acts as a receptor for adhesion proteins in the granulosa cell layer. Their interaction in granulosa cells contributes to cell survival, proliferation, and the regulation of steroid production. Although further research is warranted to determine whether ITGA6-AS directly regulates the expression of its host gene, ITGA6, this study implies that long non-coding RNAs (lncRNAs) may modulate oocyte expansion and maturation by influencing the expression of surface proteins in granulosa cells, as depicted in Fig. 3.

In a retrospective analysis comparing gene expression profiles in cumulus cells derived from high-quality embryos and poor-quality embryos, Xu and colleagues made noteworthy observations [23]. They found that the expression of lncRNA-Y00062 is markedly downregulated in cumulus cells from poor-quality embryos. This downregulation subsequently affects the expression of the gene never in mitosis gene a-related kinase 7 (NEK7), which plays a vital role in spindle assembly and mitosis. This suggests that lncRNA-Y00062 may play a crucial regulatory role in oocyte meiosis [24]. Additionally, the study identified lncRNA ENST00000502390, which exhibits high expression in cumulus cells from poor-quality embryos and is associated with elongation of very-long-chain fatty acids protein 5 (ELOVL5). ELOVL5 is involved in the biosynthesis of highly unsaturated fatty acids and plays a role in regulating oocyte maturation and ovulation processes [25]. Therefore, lncRNA ENST00000502390 may partake in oocyte maturation and ovulation by modulating the synthesis of highly unsaturated fatty acids. Another intriguing discovery centers around a novel lncRNA, AK124742, which was subsequently validated to potentially form a new lncRNA-mRNA gene pair with proteasome 26S subunit non-ATPase 6 (PSMD6) [26]. This lncRNA-mRNA pair is believed to hold significance in oocyte maturation, fertilization, embryonic development, and pregnancy processes.

Another study delves into the field of epigenetics, specifically focusing on imprinting, an essential epigenetic modification that ensures the monoallelic expression of certain genes [27]. This topic holds significant relevance as it is pivotal for the proper functioning of genes and has implications in various biological processes. The research addresses the potential impact of in vitro maturation (IVM) of oocytes on imprinting, which is an area of concern. IVM is a widely used technique in assisted reproduction, and understanding its effects on the epigenetic level is crucial for ensuring the safety and efficacy of the procedure. The study specifically investigates the methylation status of the H19 Differentially Methylated Region (DMR), a critical element in imprinting. By focusing on this region, the research provides valuable insights into the epigenetic changes that may occur during IVM. The study reveals that the methylation status of the H19 DMR is susceptible to in vitro culture conditions. This finding emphasizes the need for a thorough examination of the epigenetic consequences of IVM, especially in the context of human oocytes. The research considers oocytes at different maturation stages, including germinal vesicle (GV), metaphase I (MI), and metaphase II (MII). This comprehensive approach allows for a detailed assessment of how imprinting may be affected during various phases of oocyte development. The study's findings raise important questions about the safety and reliability of IVM as a routine procedure in assisted reproduction. The observation that some MII oocytes exhibited altered methylation patterns is particularly noteworthy, as it suggests potential risks that need further exploration. The research underscores the necessity for extended analysis, particularly focusing on MII-rescued oocytes, to fully understand the epigenetic consequences of IVM. This call for further investigation is essential in ensuring the safety and ethical practice of assisted reproductive procedures. This study addresses a critical aspect of epigenetics and assisted reproductive technology. The vulnerability of the H19 DMR to in vitro culture conditions highlights the need for rigorous assessment and extended analysis to ensure the epigenetic safety and efficacy of IVM procedures, thus contributing to the ongoing development of assisted reproductive techniques [27].

The intricate roles of lncRNAs in oocyte development and maturation are still subjects of ongoing research, but these findings provide valuable insights into the multifaceted regulatory mechanisms governing female reproductive biology. Further exploration of these mechanisms promises to advance our understanding of oocyte development and improve fertility-related interventions.

### 4.5 Participate in the formation of corpus luteum

The corpus luteum is a temporary endocrine gland formed from the remnants of the ovulated follicle. Its main function is the production of steroid hormones, and it plays a crucial role in maintaining pregnancy. Nuclear paraspeckle assembly transcript 1 (Neat1) is a nuclear-localized long non-coding RNA (lncRNA) that serves as a crucial component of nuclear paraspeckles. Although Neat1's role in regulating gene expression has been established through cell-based studies, its physiological significance has remained uncertain [28]. However, recent research has shed light on its importance in the context of the corpus luteum and pregnancy:

The corpus luteum, a transient endocrine gland derived from the remnants of the ovulated follicle, plays a pivotal role in producing steroid hormones and is essential for maintaining pregnancy. Neat1 is highly expressed in the corpus luteum, underscoring its role in this critical structure. Studies involving Neat1 gene knockout mice revealed that while these mice experienced normal ovulation, they encountered challenges in maintaining pregnancy. This unexpected fertility issue led to investigations into the underlying causes. It was observed that Neat1 gene knockout mice exhibited impaired corpus luteum function, resulting in decreased serum progesterone levels. Unilateral ovariectomy and progesterone treatment partially restored normal pregnancies in some mice. This research suggests that Neat1 is vital for corpus luteum formation and, consequently, for the successful establishment of pregnancy, especially under conditions that have yet to be precisely defined. This research elucidates the significant role of Neat1 in corpus luteum function and its impact on early pregnancy. It highlights the intricate regulatory mechanisms at play in fertility and underscores the potential influence of lncRNAs on reproductive health. Further research in this field promises to enhance our understanding of fertility and pregnancy, potentially offering new avenues for intervention and treatment.

### 4.6 Involved in the synthesis and secretion of estrogen and progesterone

Cytochrome P450 family 19 subfamily A polypeptide 1 (CYP19A1) is a pivotal member of the extensive cytochrome P450 superfamily, entrusted with the critical task of converting androgens into estrogens. Its role in estrogen synthesis is central, as estrogens are fundamental female hormones. In parallel, Cytochrome P450 family 11 subfamily A polypeptide 1 (CYP11A) serves as the rate-limiting enzyme in the conversion of cholesterol into progestins, a process that meticulously regulates the production of androgen precursors. Both CYP19A1 and CYP11A stand as indispensable components of the intricate female hormonal regulatory system.

**Fig. 4:**
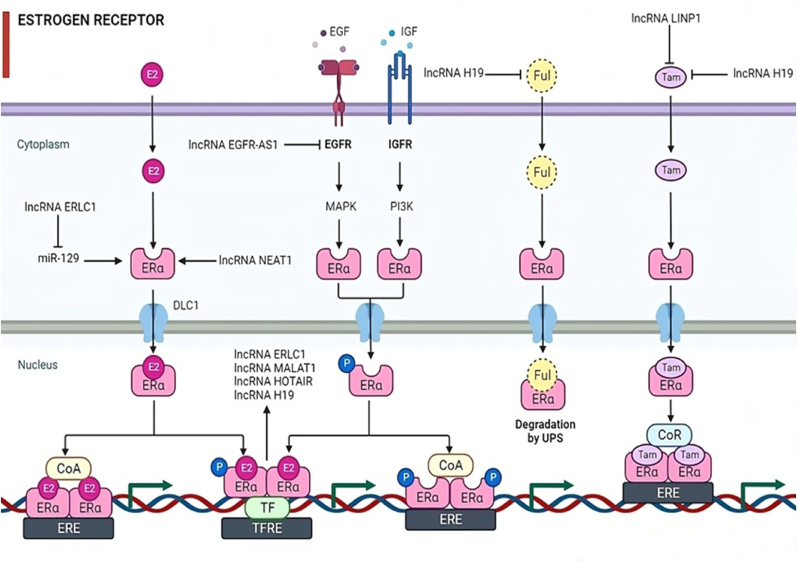
Illustration of signaling through estrogen receptors by modulating long non-coding RNAs (lncRNAs). Specifically, presented some lncRNAs lncRNA NEAT1, lncRNA H19, lncRNA MALAT1, lncRNA ERLC1, lncRNA EGFR AS1, and lncRNA HOTAIR, in estrogen signaling, emphasizing their role in physiological processes and pathogenesis of ovarian diseases such as ovarian cancer (OC). Tamoxifen (Tam) and fulvestrant (Ful) are the major drugs for patients with endocrine resistance in OC. LncRNAs (lncRNA H19 and LncRNA LINP1) have been shown to play an important role in the development of antiestrogen drug resistance. Note: ERα, Estrogen receptor alpha; CoA, Coenzyme A; ERE, Estrogen response element; TF, Transcription factor; TFRE, Transcription factor regulatory element; EGF, Epidermal growth factor; IGF, Insulin like growth factor; EGFR, Epidermal growth factor receptor; IGFR, Insulin like growth factor receptor; MAPK, Mitogen-activated protein kinase; PI3K, Phosphoinositide 3-kinase.

Recent scientific investigations have illuminated the fact that the overexpression of the long non-coding RNA Steroid Receptor RNA Activator (SRA) in the granulosa cells of mouse ovaries results in the upregulation of critical enzymes, including CYP19A1 and CYP11A1, responsible for the synthesis of sex hormones. This heightened enzyme expression leads to an increase in the levels of estradiol and progesterone, as vividly depicted in Fig. 4 [29].

Furthermore, the Steroidogenic Acute Regulatory Protein (StAR) plays a pivotal role in the transportation of cholesterol. It facilitates the movement of cholesterol from the outer mitochondrial membrane to the inner membrane in cells involved in the production of steroid hormones. This process marks the initiation of steroid hormone synthesis, serving as a pivotal and rate-limiting step in the hormonal regulation process. Recent scientific inquiries have unveiled that the long non-coding RNA H19 wields a post-transcriptional regulatory influence over StAR [30]. Experimental deletion of the H19 gene in mice solidified the conclusion that the absence of lncRNA-H19 expression results in impaired progesterone production. Mice lacking this expression displayed distinctive characteristics, including reduced size and significantly decreased litter size during their reproductive years.

In summary, CYP19A1, CYP11A, SRA, and H19 are paramount contributors in the intricate realm of hormonal regulation. They significantly impact the synthesis of estrogen, androgens, and progesterone. An exhaustive understanding of their functions and the nuances of their molecular interactions stands as a prerequisite for unraveling the intricacies of female hormonal regulation and reproductive health. This comprehensive knowledge holds the potential to provide profound insights into addressing reproductive health concerns and advancing our comprehension of the labyrinthine network of female hormonal regulation.

### 4.7 Involved in androgen synthesis

**Fig. 5:**
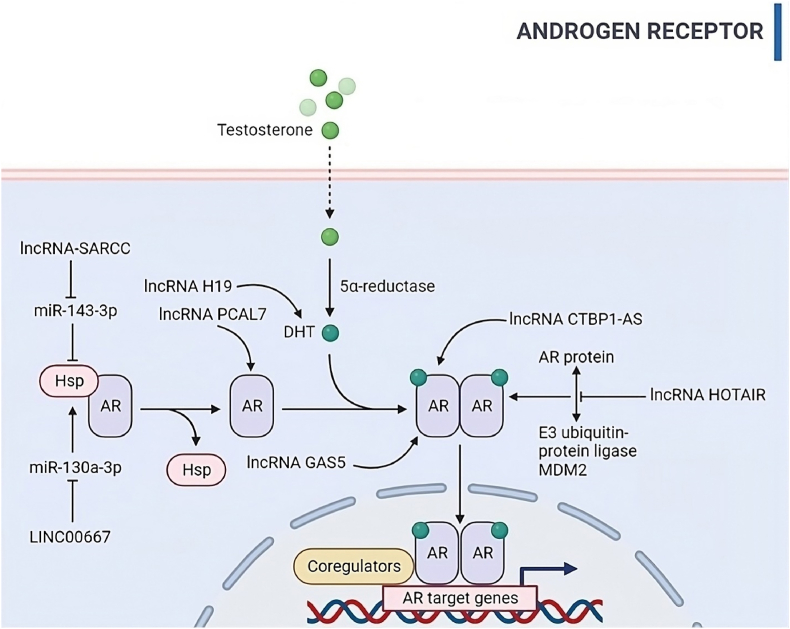
Illustration of signaling through androgen receptors by modulating long non-coding RNAs (lncRNAs). Specifically, presented some lncRNAs lncRNA-SARCC, LINC00667, lncRNA H19, lncRNA PCAL7, lncRNA GAS5, lncRNA CTBP1-AS, and lncRNA HOTAIR, in androgen signaling, emphasizing their role in physiological processes and pathogenesis of ovarian diseases such as polycystic ovary syndrome (PCOS) and ovarian cancer (OC). Note: AR, Androgen receptor; Hsp, Heat shock protein; DHT, Dihydrotestosterone.

In the human body, the enzymatic action of aromatase is responsible for converting testosterone into estradiol and dihydrotestosterone into estrone. These resultant estrogen compounds are released into both the bloodstream and follicular fluid. However, when aromatase activity is hindered or inhibited, the conversion of testosterone into estradiol is impeded, leading to an increase in androgen levels. This process is a vital component of hormonal regulation. Notably, scientific investigations have revealed a significant observation. The expression of long non-coding RNA HUPCOS in granulosa cells of individuals with polycystic ovary syndrome (PCOS) is notably elevated when compared to women with typical hormonal profiles [31]. This heightened expression of lncRNA-HUPCOS exerts a substantial influence on aromatase. To be specific, its upregulation acts to inhibit the expression of aromatase, a key enzyme in the estrogen synthesis pathway. As a result, the conversion of androgens into estrogens is impeded, leading to elevated androgen levels within the follicular fluid, as depicted in Fig. 5. In summary, the role of aromatase and the impact of lncRNA-HUPCOS on its expression constitute critical elements in hormonal regulation within the context of PCOS. The findings from this research underscore the substantial role played by lncRNAs in hormonal imbalances and their potential involvement in the pathophysiology of conditions such as PCOS. This revelation paves the way for further investigations into therapeutic interventions aimed at correcting hormonal imbalances linked to PCOS and related disorders. It underscores the intricate network of molecular interactions governing hormonal regulation within the female reproductive system.

### 4.8 Involved in the synthesis of AMH

Anti-Müllerian hormone (AMH) is a glycoprotein with critical roles in sexual development and follicular regulation. One of its primary functions is inducing the regression of the Müllerian duct during sexual differentiation. Furthermore, AMH plays a vital role in regulating the development of ovarian follicles by actively participating in the proliferation of granulosa cells [32]. Notably, during the early stages of the menstrual cycle, AMH orchestrates the recruitment, growth, development, and maturation of primordial follicles that have the lowest threshold for responding to follicle-stimulating hormone. In this intricate process, AMH acts as an inhibitor of early dominant follicles, modulating their recruitment and sensitivity to follicle-stimulating hormone [33].

Research has uncovered those mice with a knockout of the AMH gene exhibit accelerated recruitment and premature depletion of primordial follicles, leading to early ovarian failure [34]. In a related study, the deletion of the lncRNA-H19 gene in mice resulted in a substantial reduction in AMH expression. This, in turn, led to accelerated follicle recruitment, shortened developmental cycles of secondary, preantral, and antral follicles, as well as disrupted estrous cycles [35]. AMH primarily exerts its specific effects by binding to the AMH type II receptor (Amhr2). Therefore, the precise activation of the Amhr2 gene holds paramount importance for ovarian function. Emerging research suggests that lncRNA-Amhr2 is involved in the activation of the Amhr2 gene within ovarian granulosa cells. This involvement is believed to occur by enhancing the activity of the Amhr2 gene promoter and, subsequently, increasing the expression of the Amhr2 gene [36]. In summary, it is evident that lncRNAs play a pivotal role in the regulation of ovarian function by modulating the expression of AMH and influencing its effects on follicular development and the intricacies of the menstrual cycle. This insight into the interplay between lncRNAs and AMH offers promising avenues for further research and potential therapeutic interventions in the field of reproductive biology.

## 5 LNCRNAS involved in diseases related to ovarian dysfunction

### 5.1 PCOS

Polycystic ovary syndrome (PCOS) is a prevalent reproductive endocrine disorder characterized by a range of clinical manifestations. It typically presents with menstrual irregularities, which can include amenorrhea or oligomenorrhea, along with symptoms like obesity, hirsutism, acne, and, in some cases, infertility. One of its defining features is the presence of ovarian cysts [37]. To gain insights into the genetic underpinnings of PCOS, researchers have employed Weighted Gene Co-Expression Network Analysis (WGCNA) to compare gene expression variations between the ovarian granulosa cells of PCOS patients and individuals with regular hormonal profiles [38]. Their findings revealed a significant increase in the expression of lncRNA RP11151A6.4 in the ovarian granulosa cells of PCOS patients who also exhibited insulin resistance. This upregulation of lncRNA RP11151A6.4 may play a role in disrupting endocrine balance, leading to elevated androgen levels and the development of insulin resistance. Another study has shed light on the involvement of lncRNA BANCR, which is overexpressed in the granulosa cells of PCOS patients [39]. Subsequent investigations have confirmed that lncRNA BANCR initiates the apoptotic pathway, thereby inhibiting granulosa cell proliferation and promoting apoptosis. This contributes to the pathological progression of PCOS.

Another study delves into the potential therapeutic benefits of acupuncture in the context of polycystic ovary syndrome (PCOS), a complex endocrine disorder characterized by abnormal follicular development and ovulation dysfunction [40]. Acupuncture has long been suggested as a treatment for PCOS, but this study seeks to shed light on the underlying molecular mechanisms that may explain its efficacy. The study sets the stage by emphasizing the significance of PCOS, an enigmatic condition that affects many women and leads to issues such as abnormal follicular development and ovulation dysfunction. It underscores the need for effective treatments that target the root causes of these problems. The research rightly acknowledges acupuncture as a potential therapeutic intervention for PCOS. While its benefits have been observed in clinical settings, the underlying mechanisms have remained unclear until this study. The study's primary hypothesis is that acupuncture's benefits may be linked to its effects on long non-coding RNA LncMEG3 and its modulation of the PI3K/AKT/mTOR pathway. By examining these molecular pathways, the research aims to provide a more comprehensive understanding of how acupuncture affects PCOS. To test their hypothesis, the study employs a rat model of PCOS and utilizes a combination of acupuncture points. They then conduct a comprehensive analysis, including histological examination, hormone level assessments, and molecular analyses. The results are promising, indicating that acupuncture leads to improvements in ovarian morphology, hormonal balance, and granulosa cell activities. Acupuncture downregulates the expression of LncMEG3, subsequently inhibiting the PI3K/AKT/mTOR pathway. This, in turn, reduces the autophagy of granulosa cells and normalizes their proliferation, thereby addressing the root causes of PCOS-related ovarian dysfunction. The study's findings offer significant clinical potential for acupuncture as a safe and effective treatment for PCOS-related ovulatory dysfunction [40]. By targeting the molecular pathways underlying PCOS, acupuncture may provide an innovative approach to address this complex condition. This study provides a valuable contribution to the understanding of how acupuncture can effectively address PCOS-related ovarian dysfunction at a molecular level. Its findings offer hope for more targeted and personalized treatment options for women affected by this challenging condition.

Chen et al. explores the potential benefits of acupuncture as a treatment for polycystic ovary syndrome (PCOS), a complex condition characterized by abnormal follicular development and ovulatory dysfunction [41]. The research focuses on unraveling the molecular mechanisms underlying the effects of acupuncture in addressing these issues. The study underscores the importance of PCOS as a prevalent condition affecting women, particularly its manifestations related to ovarian follicular development and ovulation. It highlights the need for effective interventions to address the core issues of PCOS. Acupuncture has long been considered as a potential treatment for PCOS, and this study aims to shed light on the mechanisms by which acupuncture exerts its effects. It delves into the molecular underpinnings of acupuncture in the context of PCOS. The study's primary hypothesis is that acupuncture's benefits may be mediated through the regulation of long non-coding RNA LncMEG3. LncMEG3 is proposed to act as a key player in inhibiting granulosa cell apoptosis in PCOS patients. The researchers use a PCOS-like rat model induced by dehydroepiandrosterone (DHEA) and apply acupuncture to the rats for a defined period. The study encompasses a range of analyses, including ovarian morphology assessments, sex hormone measurements, and evaluations of granulosa cell activities. The results of the study are promising. It reveals that LncMEG3 and miR-21-3p play crucial roles in the development of PCOS. Silencing LncMEG3 leads to improvements in sex hormone regulation, ovarian histopathology, and granulosa cell viability and number. Importantly, it also inhibits granulosa cell apoptosis, contributing to the normalization of follicular development. Acupuncture is shown to improve ovarian morphology and hormone levels in PCOS rats. It enhances the viability and number of granulosa cells while also inhibiting granulosa cell apoptosis. This effect is achieved by targeting miR-21-3p via LncMEG3. The study's findings open exciting possibilities for acupuncture as a safe and effective treatment for addressing the follicular developmental abnormalities seen in PCOS [41]. By targeting specific molecular pathways, acupuncture offers a novel approach to addressing this multifaceted condition. This research provides valuable insights into the potential role of acupuncture in ameliorating follicular development abnormalities in PCOS. It highlights the relevance of molecular mechanisms, such as LncMEG3 and miR-21-3p, in acupuncture's therapeutic effects. The results offer hope for more targeted and personalized treatments for women affected by PCOS, emphasizing the importance of understanding the molecular underpinnings of this complex condition.

In essence, the study of lncRNAs offers a fresh perspective on our understanding of the complex clinical landscape of PCOS. It provides opportunities for exploring novel therapeutic approaches to address this multifaceted condition, offering hope for more effective clinical treatments.

### 5.2 Premature ovarian insufficiency

Premature ovarian insufficiency (POI) is a condition characterized by the decline in ovarian function that occurs in women before the age of 40. It is primarily identified by irregular menstrual cycles or even complete amenorrhea, along with elevated levels of gonadotropins (>25 U/L) and fluctuating estrogen levels [42]. Beyond these immediate symptoms, POI is associated with long-term risks, including osteoporosis and cardiovascular diseases, which significantly impact the quality of life for affected individuals [43]. In significant research finding by Yao et al., an extensive analysis of RNA transcriptomes in ovarian cortical tissues and serum samples from both POI patients and healthy individuals revealed distinct alterations in the expression of specific long non-coding RNAs (lncRNAs) [44]. This investigation unveiled that lncRNA-ADAMTS1-1꞉1 and lncRNA-PHLDA3-3꞉2 were significantly upregulated in POI patients, while lncRNA-SAMD14-5꞉3, lncRNA-COL1A1-5꞉1, and lncRNA-GULP1-2꞉1 displayed marked downregulation. These serological examinations were consistent with the analysis of ovarian cortical tissues. Further validation confirmed a positive correlation between lncRNA-GULP1-2꞉1 and its potential target genes in ovarian tissue, all of which were notably downregulated. Another study delved into the influence of lncRNA-Meg3, showing a substantial increase in its expression in mouse ovarian tissue following cyclophosphamide treatment [45]. In vitro experiments involving isolated and cultured mouse ovarian granulosa cells provided insights into the underlying mechanism. lncRNA-Meg3 was found to hinder cell proliferation by activating the p53-p66Shc pathway and increasing the expression of the apoptosis-related protein caspase-3. These effects ultimately led to ovarian insufficiency. These research findings illuminate the intricate molecular interactions that underlie POI and highlight the pivotal role of lncRNAs in this context. Ongoing research in this field holds the potential to deepen our understanding of POI and may contribute to the development of more effective diagnostic and therapeutic approaches for this condition. Such advances are promising for improving the quality of life for individuals affected by POI and for advancing women's reproductive health.

## 6 Correlation between LNCRNA as CERNA and ovarian function

In addition to their direct involvement in the regulation of gene expression through mechanisms such as DNA methylation, histone modifications, and chromatin remodeling, long non-coding RNAs (lncRNAs) can also function as competing endogenous RNAs (ceRNAs). They serve as competitive binding partners for microRNAs (miRNAs), thus diminishing the silencing impact of miRNAs on their target mRNAs. This intricate interplay forms a complex network known as the lncRNA/miRNA/mRNA network. In the context of ovarian function, lncRNAs operating as ceRNAs introduce a fresh perspective, providing a more comprehensive understanding of the molecular mechanisms that underlie ovarian function. One such lncRNA, known as HOX transcript antisense RNA (HOTAIR), is an antisense gene RNA situated within the genomic region between the human HOXC11 and HOXC12 homologous clusters [46]. Previous research has hinted at the potential of lncRNA-HOTAIR overexpression to induce apoptosis in ovarian granulosa cells, consequently affecting ovarian function [47]. However, most investigations into lncRNA-HOTAIR as a ceRNA have been concentrated in the domain of cancer. Recent research focusing on ovarian function uncovered that lncRNA-HOTAIR exhibited overexpression in ovarian granulosa cells of a rat model with polycystic ovary syndrome (PCOS) [48]. Furthermore, it was verified that lncRNA-HOTAIR, by competitively binding to miR-130a, upregulated the expression of insulin-like growth factor 1 in PCOS rats. This activity exacerbated endocrine disruption, resulted in reduced granulosa cell proliferation, and promoted cell apoptosis. These compelling findings indicate that lncRNA-HOTAIR holds the potential to emerge as a novel target for molecular therapy in addressing PCOS. LncRNAs acting as ceRNAs have introduced a new layer of complexity to our understanding of ovarian function by revealing their intricate interactions within the lncRNA/miRNA/mRNA network. Among these, lncRNA-HOTAIR's implications in PCOS underscore the potential for innovative therapeutic strategies, offering hope for addressing endocrine disorders and improving ovarian health.

LncRNA-PWRN2, also known as Proder-Willi region non-protein coding RNA 2, is a fascinating lncRNA with intriguing implications for PCOS [49]. While initially identified as primarily expressed in testes, recent research has unveiled its overexpression in PCOS patients. The significance lies in its function as a competing endogenous RNA (ceRNA). In this role, lncRNA-PWRN2 competitively binds to miR-92b-3p, a microRNA, leading to the upregulation of transmembrane protein 120B (TMEM120B) [50]. TMEM120B, in turn, is associated with fat formation. Elevated TMEM120B levels can induce obesity, disrupt oocyte maturation, and have adverse effects on ovarian function. This discovery indicates that lncRNA-PWRN2 might be a promising therapeutic target for addressing PCOS, offering hope for potential interventions to mitigate the complex manifestations of the syndrome. In PCOS, the intricate involvement of lncRNAs in disease pathogenesis is further exemplified by lncRNA CD36-005 [51]. This lncRNA displays significant upregulation in the uterine tissues of PCOS rat models. However, it takes on a contrasting role during early pregnancy in rats, where it experiences substantial downregulation. The significance of lncRNA CD36-005 in PCOS appears to revolve around its influence on the pathogenesis. It targets interferon-induced gene S-adenosylhomocysteine hydrolase domain-containing protein 2, thus inhibiting endometrial decidualization [52]. This inhibition has considerable implications for pregnancy and reproductive health. In a comprehensive study conducted by Fu et al., 2,147 differentially expressed mRNAs, 158 lncRNAs, and several miRNAs were identified in a PCOS rat model [53]. Among them, lncRNAs RT1-M3-1-002 and CD36-005, along with several miRNAs and their respective target genes, were discovered. The intricate lncRNA/miRNA/mRNA network indicates that lncRNAs RT1-M3-1-002 and CD36-005 might play vital roles in the pathogenesis of PCOS. They do so by competitively binding to miR-146a-5p and miR-448-5p, thus inhibiting these microRNAs' negative regulation of target genes Csmd1 and Ltbp4. This finding emphasizes the complex web of interactions that underlie PCOS pathogenesis, underscoring the need for further experimental validation to elucidate the specific functions of lncRNA RT1-M3-1-002 in this context.

LncRNAs such as lncRNA-PWRN2 and lncRNA CD36-005 are shedding light on the multifaceted landscape of PCOS. Their intricate regulatory roles, their competitive interactions with microRNAs, and their impact on genes associated with obesity, oocyte maturation, and endometrial decidualization signify their potential as crucial players in understanding and potentially treating PCOS. This novel perspective on the involvement of lncRNAs in PCOS opens exciting avenues for future research, aiming to unravel the intricate mechanisms of this complex syndrome and, ultimately, improve the lives of individuals affected by PCOS.

The role of long non-coding RNAs (lncRNAs) like Gm2044 and GULP1-2:1 in the realm of reproductive health, particularly within the context of ovarian function, is an area of growing significance and scientific exploration [54,55]. LncRNA Gm2044 has garnered attention due to its substantial involvement in male reproductive processes, and it's noteworthy that this lncRNA is abundantly expressed in both testicular and ovarian tissues. However, the research exploring its functions in ovarian processes is relatively limited [56]. An insightful study has offered valuable findings regarding the potential impact of lncRNA Gm2044 on ovarian function. It was discerned that lncRNA Gm2044 operates as a competing endogenous RNA (ceRNA) by competitively binding to miR-138-5p. This interaction, in turn, results in the upregulation of nuclear receptor subfamily 5 group A member 1 (Nr5a1), a pivotal transcription factor closely involved in the regulation of ovarian steroidogenesis. In practical terms, this signifies that lncRNA Gm2044 facilitates the production of 17β-estradiol (E2), a crucial ovarian steroid hormone. Dysregulation of lncRNA Gm2044 may have the potential to contribute to the pathogenesis of conditions like polycystic ovary syndrome (PCOS) by impacting the production of ovarian steroid hormones. This insight underscores the critical importance of comprehending the intricate regulatory roles of lncRNAs within the realm of ovarian function.

LncRNA GULP1-2:1 has received limited attention in scientific research, particularly within the context of ovarian function [57]. Nevertheless, one notable study has started shedding light on its potential involvement in reproductive health, particularly in the context of premature ovarian failure (POF). The study involved a comparative analysis of the transcriptome levels of granulosa cells derived from patients with POF and individuals with typical ovarian function. The results unveiled significant differential expression of various molecules, including lncRNAs, miRNAs, and mRNAs, within the ovarian tissue of POF patients. Furthermore, subsequent research validated the potential influence of lnc-GULP1-2:1 on the regulatory role of miR-204-5p concerning the collagen type III alpha 1 (COL3A1) gene. By interfering with estrogen production, granulosa cell proliferation, and follicle growth, lnc-GULP1-2:1 may have a role in contributing to the development of POF. This insight illuminates the intricate network of molecular interactions involved in ovarian function and underscores the potential significance of lncRNAs in the realm of reproductive disorders. In summary, lncRNAs like Gm2044 and GULP1-2:1 is gradually emerging as key players in the realm of ovarian function and reproductive health. Their ability to engage in competitive interactions with microRNAs and influence crucial genes associated with steroidogenesis and ovarian development highlights the complexity of the molecular mechanisms involved in reproductive disorders. Further research in this field is of utmost importance to broaden our understanding and potentially identify novel therapeutic targets for conditions such as PCOS and POF.

LncRNA-H19, a well-studied long non-coding RNA, is located on human chromosome 11q15.5 and comprises around 2,300 nucleotides [58]. Extensive research has emphasized its aberrant expression patterns in the context of various conditions, including polycystic ovary syndrome (PCOS), infertility, and ovarian dysfunction [[59], [60], [61]]. It's also been associated with the regulation of anti-Müllerian hormone (AMH) and progesterone expression levels. Despite these findings, investigations into lncRNA-H19's role as a competing endogenous RNA (ceRNA) in ovarian function and related diseases have remained somewhat limited. In a notable study, alterations in the expression levels of lncRNA-H19 and miRNA-29a were observed in the peripheral blood of PCOS patients [62]. Leveraging bioinformatics methodologies, this research identified the androgen receptor (AR) and insulin-like growth factor-1 (IGF-1) as likely target genes for miRNA-29a. Subsequently, the study validated the binding capacity of lncRNA-H19 with miRNA-29a and miRNA-29a with AR and IGF-1 in ovarian granulosa cells. The study's results unveiled that the elevated expression of lncRNA-H19 in PCOS patients may function as a competitive “sponge,” binding to miRNA-29a. This interaction leads to the overexpression of the target genes AR and IGF-1 proteins, ultimately resulting in increased androgen levels. This, in turn, actively contributes to the pathogenesis of PCOS. These findings provide insights into one of the mechanisms through which lncRNA-H19 affects the intricacies of PCOS at the molecular level.

LncRNA-H19's multifaceted roles in ovarian function and its associations with diseases such as PCOS continue to be a subject of growing interest. This study has shed light on a specific mechanism through which lncRNA-H19 can impact PCOS pathogenesis, offering potential therapeutic implications. Further exploration of lncRNA-H19's functions in ovarian health and disease promises to deepen our understanding of these intricate molecular processes.

LncRNA-Metastasis-associated lung adenocarcinoma transcript 1 (MALAT1), an 8.5-kilobase long non-coding RNA located on chromosome 11q13, has been extensively studied, primarily in the context of non-small cell lung cancer (NSCLC) [63]. However, its relevance extends to various other health and disease contexts, including breast cancer, colorectal cancer, and ovarian cancer [[64], [65], [66]]. Within the realm of the reproductive system, MALAT1 has emerged as a significant player, impacting pregnancy loss and endometriosis [67]. In the context of ovarian function, research has unveiled intriguing insights by examining patients with polycystic ovary syndrome (PCOS). This study revealed decreased expression of lncRNA-MALAT1 in PCOS patients, accompanied by increased levels of miR-125b and miR-203a [68]. Subsequent investigations confirmed that lncRNA-MALAT1, operating as a competing endogenous RNA (ceRNA), competes for binding with miR-125b and miR-203a. This interaction results in the downregulation of transforming growth factor beta receptor 1 (TGFBR1) and transforming growth factor beta receptor 2 (TGFBR2) expression. This regulatory mechanism is believed to influence granulosa cell proliferation and apoptosis, actively contributing to the complex pathological and physiological processes in PCOS. These findings suggest that lncRNA-MALAT1 has the potential to serve as a novel molecular target for the diagnosis and treatment of PCOS.

MALAT1's diverse roles in various disease contexts, including PCOS, highlight its potential as a promising molecular target for advancing our understanding and management of reproductive disorders. Further research into the multifaceted functions of MALAT1 in ovarian health and disease is warranted to unlock its full therapeutic potential.

## 7 Conclusion

Long non-coding RNAs (lncRNAs) constitute a diverse group of genetic molecules distributed extensively across the human genome [[69], [70], [71], [72]]. These molecules exhibit a remarkable range of variations in their types and the diverse mechanisms through which they exert their influence. This article delves deep into the intricate and multifaceted role that lncRNAs play in orchestrating critical facets of ovarian function, encompassing processes as intricate and crucial as follicular growth, ovarian developmental processes, and sex hormone synthesis. LncRNAs' uniqueness in this context lies in their role as competitive endogenous RNAs (ceRNAs), a central component of post-transcriptional regulation. The primary focus of this investigation centers on how lncRNAs, by operating as ceRNAs, contribute to the finely tuned regulation of ovarian function and, intriguingly, their involvement in the development of disorders and conditions associated with ovarian dysfunction. However, it's essential to emphasize that the concept of ceRNA interactions represents a complex and far-reaching system of RNA-mediated transcriptional control. Our current comprehension of ceRNA regulatory networks is still in its early stages, with the majority of research concentrated on contexts related to cancer. While a substantial body of evidence has convincingly demonstrated the ability of lncRNAs to serve as ceRNAs, thereby contributing to the maintenance of normal ovarian function and offering potential as innovative targets for diagnosing and treating ovarian-related conditions, it's vital to acknowledge that the ceRNA hypothesis remains a topic of ongoing debate. Its validity necessitates further substantiation through extensive studies involving large and diverse populations. Hence, there is a pressing and compelling need for further exploration and in-depth investigations to attain a comprehensive understanding of the multifaceted role of lncRNAs as ceRNAs within the intricate landscape of ovarian function and the disorders associated with it. This pursuit is critical for advancing our knowledge and potentially paving the way for novel therapeutic strategies in the realm of reproductive health.

In summary, long non-coding RNAs (lncRNAs) are gradually emerging as pivotal regulators in the intricate machinery of ovarian function. Their influence extends to essential processes such as follicular growth, ovarian development, and sex hormone synthesis. Through their roles as competitive endogenous RNAs (ceRNAs), lncRNAs demonstrate a remarkable ability to intricately modulate gene expression and control the signaling pathways that are indispensable for maintaining normal ovarian function. However, it is essential to acknowledge that the ceRNA hypothesis and its precise relevance to ovarian function and disorders related to it remain subjects that warrant closer and more comprehensive examination. The relentless pursuit of knowledge in this field holds the potential to uncover novel diagnostic markers and therapeutic targets that can profoundly impact and improve women's health, particularly in the context of reproductive well-being. Further research and in-depth investigations are needed to fully understand the multifaceted role of lncRNAs as ceRNAs within the intricate landscape of ovarian function and associated disorders. This ongoing exploration is crucial for advancing our understanding and improving the diagnosis and treatment of conditions related to ovarian dysfunction.

## Funding

Central Hospital Affiliated to 10.13039/501100004867Chongqing University of Technology, The Science and Technology Bureau of Banan District, Chongqing [grant number 2021-36]. Central Hospital Affiliated to Chongqing University of Technology, Gonglian yicun No.1 street lijiatuo, Banan district, Chongqing 400054, P.R. China.

## CRediT authorship contribution statement

**Muhammad Usman:** Writing – review & editing, Writing – original draft. **Ai Li:** Methodology, Investigation. **Dan Wu:** Software, Project administration. **Yang Qinyan:** Formal analysis, Data curation. **Lin Xiao Yi:** Formal analysis, Data curation. **Guiqiong He:** Visualization, Validation, Supervision, Project administration. **Hong Lu:** Supervision, Project administration, Funding acquisition.

## Declaration of competing interest

All authors declare that there are no competing interests.
